# Differences in Inflammatory Response Induced by Two Representatives of Clades of the Pandemic ST258 *Klebsiella pneumoniae* Clonal Lineage Producing KPC-Type Carbapenemases

**DOI:** 10.1371/journal.pone.0170125

**Published:** 2017-01-12

**Authors:** Giuseppe Castronovo, Ann Maria Clemente, Alberto Antonelli, Marco Maria D’Andrea, Michele Tanturli, Eloisa Perissi, Sara Paccosi, Astrid Parenti, Federico Cozzolino, Gian Maria Rossolini, Maria Gabriella Torcia

**Affiliations:** 1 Department of Experimental and Clinical Medicine, University of Firenze, Firenze, Italy; 2 Department of Medical Biotechnologies, University of Siena, Siena, Italy; 3 Department of Experimental and Clinical Biomedical Sciences, University of Firenze, Firenze, Italy; 4 Department of Health Sciences, University of Firenze, Firenze, Italy; 5 Clinical Microbiology and Virology Unit, Careggi University Hospital, Firenze, Italy; 6 IRCCS Don Carlo Gnocchi Foundation, Firenze, Italy; Louisiana State University, UNITED STATES

## Abstract

ST258-*K*. *pneumoniae* (ST258-KP) strains, the most widespread multidrug-resistant hospital-acquired pathogens, belong to at least two clades differing in a 215 Kb genomic region that includes the cluster of capsule genes. To investigate the effects of the different capsular phenotype on host-pathogen interactions, we studied representatives of ST258-KP clades, KKBO-1 and KK207-1, for their ability to activate monocytes and myeloid dendritic cells from human immune competent hosts. The two ST258-KP strains strongly induced the production of inflammatory cytokines. Significant differences between the strains were found in their ability to induce the production of IL-1β: KK207-1/clade I was much less effective than KKBO-1/clade II in inducing IL-1β production by monocytes and dendritic cells. The activation of NLRP3 inflammasome pathway by live cells and/or purified capsular polysaccharides was studied in monocytes and dendritic cells. We found that glibenclamide, a NLRP3 inhibitor, inhibits more than 90% of the production of mature IL-1β induced by KKBO1 and KK207-1. KK207-1 was always less efficient compared to KKBO-1 in: a) inducing NLRP3 and pro-IL-1β gene and protein expression; b) in inducing caspase-1 activation and pro-IL-1β cleavage. Capsular composition may play a role in the differential inflammatory response induced by the ST258-KP strains since capsular polysaccharides purified from bacterial cells affect NLRP3 and pro-IL-1β gene expression through p38MAPK- and NF-κB-mediated pathways. In each of these functions, capsular polysaccharides from KK207-1 were significantly less efficient compared to those purified from KKBO-1. On the whole, our data suggest that the change in capsular phenotype may help bacterial cells of clade I to partially escape innate immune recognition and IL-1β-mediated inflammation.

## Introduction

Carbapenem-resistant *Enterobacteriaceae*, and especially *Klebsiella pneumoniae* (KP) producing the KPC-type carbapenemases (KPC-KP), have emerged as an important cause of healthcare-associated infections correlated with high morbidity and mortality [1–3]. The pandemic diffusion of KPC-KP has largely been contributed by the clonal expansion of strains belonging to sequence type (ST) 258 (ST258) producing either the KPC-2 or the KPC-3 carbapenemases [4–10].

The capsular diversity and molecular evolutionary history of ST258-KP has recently been described [4,11–14] suggesting that large genomic recombination events were responsible for the emerging of clonal and subclonal lineages.

Briefly, ST258-KP descended from an ST11-like ancestor which acquired a 1.1 Mb genomic region including the *cps* locus (*cps*_BO-4_) from ST442-like *K*. *pneumoniae* via recombination [4,13,14]. This clone has been named ST258 clade II/ST258-2 [4,11] or ST258b [14]. Subsequently a third *cps* (*cps*_207-2_) locus was acquired forming a new sublineage of ST258 named ST258 clade I/ST258-1 [4] or ST258a [14]. The effects of genomic variations within the clonal complex, including the change in capsular phenotype (capsular switch), on the host-pathogen interactions are not defined yet. Recently it has been reported that the uptake of ST258-KP strains by activated neutrophils *in vitro* is very low at least compared to *Staphylococcus aureus*, used as positive control in the assay [15]. These data suggest the resistance of ST258-KP strains to neutrophil phagocytosis but they do not correlate the capsular phenotype to this virulence trait [11].

Although neutrophil functions are critical to clear infections sustained by *K*. *pneumoniae*, other innate immune cells such as monocyte/macrophages and dendritic cells also participate in bacterial clearance by producing cytokines and inflammatory mediators involved in the recruitment of neutrophils, as well as in T-cell activation and differentiation [16]. It is commonly accepted that a major factor surrounding the ability of *K*. *pneumoniae* to persist at mucosal level is their failure to stimulate the robust pro-inflammatory response.

To investigate whether the phenotypic features acquired by ST258-KP strains in the course of clonal evolution differently affect the human immune-inflammatory response we used strains of ST258-KPC-KP representatives of the two clades and studied their ability to induce pro-inflammatory pathways in Myeloid Dendritic Cells (MDC) and monocytes. The strain KK207-1 was used as representative of clade I (or clade a/A), the strain KKBO-1 was used as representative of clade II (or clade b/B).

## Materials and Methods

### Antibodies and reagents

Antibodies to cleaved caspase-1, phospho-p38MAPK, were from Cell Signaling Technology (Danvers, MA, USA). Antibodies to phosphorylated NF-κB, α-Tubulin, IL-1β, were purchased from Santa Cruz Biotechnology, (Santa Cruz, CA, USA). Antibodies to human NLRP3 and NLRC4 were purchased from Cell Signaling Technology. Anti-human CD14-coniugated microbeads were from Miltenyi Biotec (Bergisch Gladbach, Germany). Limulus Assay was purchased from Cape Cod Incorporated (Falmouth, MA, USA).

Glibenclamide was from Sigma Aldrich (Saint Louis, MO, USA). LPS was purchased from Invitrogen (Carlsbad, CA, USA). RPMI 1640, antibiotics (penicillin/streptomycin), L-glutamine, heat-inactivated FBS were purchased from Euroclone (Pero, Italy) and used for cultures with dendritic cells.

### ST258 *K*. *pneumoniae* strains: properties and culture methods

Six strains of KPC-KP were selected. The sequence type and capsular phenotype and the reference clade of the selected strains are reported on Table 1.

**Table 1:** Characteristics of the *K*. *pneumoniae* strains selected in this study.

Strain	ST	CPS	Clade	Reference	Accession no.
KKBO-1	258	CPS_BO4_	2	[17]	AVFC00000000
KK207-1	258	CPS_207-2_	1	[13]	LJOO00000000
FIPP-1	258	CPS_BO4_	2	[18]	-
01C03	258	CPS_207-2_	1	[19]	-
06C07	258	CPS_207-2_	1	[19]	-
MR190	512	CPS_BO4_	2	-	-

All strains were grown overnight in Mueller Hinton Broth (MHB), diluted 1:100 in the same medium and incubated again at 37°C until an OD_600_ of 0.3 was reached. Bacterial cells were UV-treated (20 minutes at 1 J/cm^2^) and used as stimulus for the production of inflammatory cytokines by monocytes and MDC as reported in the specific section.

KKBO-1and KK207-1, isolated in 2010 from two different Italian hospitals and epidemiologically unrelated to each other, were used in all of the experiments as representative strains of clade 2 and clade 1 respectively [13,17].

PFGE (Pulsed-field gel electrophoresis) analysis demonstrated that the selected strains are representative of the major pulsotypes of ST258 KPC-KP clones from the Italian epidemic occurred in 2011 [13,17,19]. Remarkably, very similar pulsotypes were recently observed for *K*. *pneumoniae* isolates included in a recent surveillance study conducted in Italian hospitals, suggesting the persistence of the two clonal lineages in our country [20].

Moreover, both isolates were positive for the operon required for galactan-III synthesis that was recently described in the majority of ST258 clinical isolates [21].

Where indicated, bacterial cells were UV-treated (20 minutes at 1 J/cm^2^) and used as antigen source at the reported concentrations. Cultures of monocytes or MDC with live bacterial cells were performed at 1:1 cell ratio for 4–7 hours. Cultures of monocytes or MDC with UV-treated bacterial cells were performed at 1:10 cell ratio for the times indicated.

### Endocytosis of bacterial cells

To determine the rate of bacterial endocytosis monocytes and MDC were cultured with live bacterial cells at 1:1 cell ratio for 2 hours. At the end of incubation, cells were washed and lysed with 0.1% triton X-100; the non-endocytosed bacterial cells were plated on MHA for CFU counts. Based on CFU count after an overnight growth, we calculated that ≈40% ± 4 and ≈53% ± 5 of bacterial cells were endocytosed by monocytes and MDC, respectively. No differences emerged between KKBO-1 and KK207-1.

### Bacterial capsular polysaccharide (CPS) extraction and purification

Bacterial cells grown overnight as reported above were vortexed for 4 hours at 4°C and centrifuged at 10,000X*g* for 15 min. The supernatant was collected, mixed with 3 volumes of acetone and incubated overnight at 4°C. Samples were then centrifuged at 10,000X*g* for 10 min. The pellet was washed and treated with RNAse (Qiagen, Hilden, Germany), DNAase (Promega, Madison, WI, USA) and proteinase K (Qiagen, Hilden, Germany). Then each sample was dialyzed against PBS and the LPS was removed by centrifugation at 105,000Xg for 8 hours at 15°C (modified from [22]).

The amounts of bacterial capsule were measured as previously described [23]. Briefly, 50 μl of sample or standard were mixed with 200 μl of 25 mM sodium tetraborate in sulfuric acid in a 96 multiwell plate. The plate was incubated at 100°C for 15 minutes, and at room temperature for 15 minutes. 50 μl of 0.125% carbazole in absolute ethanol were added to each well, and the plate was incubated at 100°C for 10 min and finally maintained at room temperature for 15 minutes. Plates were analysed at 550 nm using VICTOR microplate reader (Perkin Elmer, Waltham, MA, USA). Endotoxin contamination, assessed by limulus assay, was not revealed in capsular preparation (1–20 μg/ml). Purified capsular components were used as stimulus at the concentration of 5 μg/ml.

### Preparation, isolation and culture of cells

Buffy coats from 6 healthy donors were supplied by Transfusional Center of Azienda Ospedaliera Careggi (Firenze, Italy). CD14^+^ cells were isolated using anti-CD14 conjugated microbeads (Miltenyi Biotec, Bergisch Gladbach, Germany). To obtain MDC CD14^+^ cells were cultured in the presence of 20 ng/ml of human recombinant (hr) IL-4 and 50 ng/ml of hr Granulocyte Macrophage Colony Stimulating Factor GMCSF (R&D Systems, Minneapolis, MN, USA) for seven days at 37°C in a humidified chamber with 5% CO_2_. MDC were recovered, plated at 10^6^ cells/ml and stimulated with bacterial cells. Monocytes and MDC were cultured with live bacterial cells: 1:1 for 4–7 hours or with UV-inactivated bacterial cells 1:10 for the indicated times.

### Flow cytometry

5x10^5^ MDC or monocytes, cultured for 16 hours with live bacterial cells were stained with a propidium iodide (Roche) for 15 min, washed with cold PBS and analysed by ACCURI instrument (BD Biosciences, Franklin Lakes, NJ, USA). Data were analysed by CflowPlus software (BD Biosciences, Franklin Lakes, NJ, USA). Ten thousand events for each sample were acquired.

### LDH release

LDH activity released from damaged cells was revealed in culture supernatants using the Cytotoxicity Detection Kit^PLUS^ (LDH) (Sigma Aldrich) according to manufacturer’s instructions.

### Western blot analysis

Detection of mature IL-1β, NLRP3, NLRC4 and, cleaved Caspase 1: 2x10^6^ MDC or monocytes were cultured in complete medium for 7 hours in the presence or absence of live bacterial cells (cell ratio 1:1) or of 5 μg/ml purified capsular preparation.

Detection of phospho-p38MAPK and phospho-NF-κB: 2x10^6^ MDC or monocytes were cultured in complete medium in the presence or absence of live bacterial cells (cell ratio 1:1) or of 5 μg/ml purified capsular preparation for 45 minutes.

Cells were then lysed with RIPA buffer (50 mM Tris-HCl, pH 7.4; 150 mM NaCl; 2 mM EDTA; 1mM NaF; 1 mM sodium orthovanadate, 1% NP-40) in the presence of phosphatase inhibitor cocktail 2 and 3, protease inhibitor cocktail (Sigma Aldrich, Saint Louis, MO, USA) and centrifuged at 12,000X*g* for 15 minutes. 40 μg of proteins were loaded onto SDS-PAGE and blotted onto nitrocellulose filters (GE Healthcare, Fairfield, CT, USA). Membranes were stained with rabbit anti-caspase 1 (Cell Signaling Technology, Danvers, MA, USA) mouse anti-α-Tubulin, rabbit anti-IL-1β, rabbit anti-NF-κB (Santa Cruz Biotechnology, Santa Cruz, CA, USA) and rabbit anti-phospho-p38 MAPK (Cell Signaling Technology, Danvers, MA, USA) antibodies, 1:1,000 final dilution. Anti-rabbit IgG (H+L) DyLight800 or anti-mouse IgG (H+L) DyLight 650 (Thermo Fisher, Waltham, MA, USA) were used as secondary antibodies at 1:10,000 final dilution. The reactions were visualized and acquired by the Licor^®^Odyssey Infrared Imaging System (LI-COR Biosciences, Lincoln, NE, USA).

### Cytokine measurement

IL-1β, IL-6, TNFα, concentrations in culture supernatants of MDC or monocytes were determined as previously described [24] by using Milliplex kit (HCYTOMAG-60K Merk KGaA, Darmstadt, Germany) and BIOPLEX (Bio-Rad, Hercules, CA, USA) apparatus according to the manufacturer's recommendations.

### RT-PCR

Cellular RNA was extracted using TRIzol® Reagent (Invitrogen, Carlsbad, CA, USA) according to the manufacturer's recommendation and reverse transcription of mRNA was performed using High-Capacity cDNA Reverse Transcription Kit (Applied Biosystems, Foster City, CA, USA). Amplifications process was performed with RT2 Real-Time™ SYBR Green / ROX PCR Master Mix (Applied Biosystems, Foster City, CA, USA) according to manufacturer's recommendations on an ABI PRISM 7900. Data were normalized to the mean value of house-keeping gene GAPDH mRNA and the relative amount of mRNA was calculated by using the 2^-ΔCT^ method (36).

The nucleotide sequences of PCR Forward (Fw) and Reverse (Rv) primers are:

NLRP3: Fw 5'-GAGGCAACACTCTCGGAGAC-3',

Rv 5'-TCTGGCTGGAGGTCAGAAGT-3';

Pro-IL-1β: Fw 5'-TCCAGGGACAGGATATGGAG-3',

Rv 5'-TCTTTCAACACGCAGGACAG-3';

GAPDH: Fw 5'-CACCATCTTCCAGGAGCGAG-3',

Rv 5'-AAATGAGCCCCAGCCTTCTC-3';

### Statistical analysis

Statistical analysis was performed by one-way ANOVA and Student *t*-test. p ≤ 0.05 was considered significant.

## Results

### Effects of ST258 *K*. *pneumoniae* isolates of the two clades on cytokine production by monocytes and immature MDC

**Fig 1:**
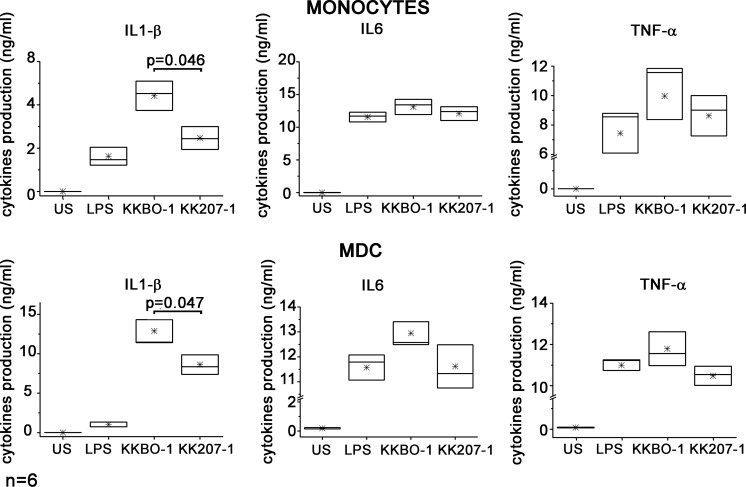
Effect of ST258 KP strains on inflammatory cytokines production by monocytes and MDC. Monocytes or MDC were cultured at 10^6^ cells/ml in RPMI with 10% FBS in the presence or absence (US) of UV-inactivated ST258-KP strains at 1:10 ratio or of LPS (400 ng/ml). Conditioned media were collected after 16 hours of culture. Cytokine production was measured by Immunoplex array. Box-chart plots show cytokine production from 6 different healthy donors. The boxes extend from SE; the horizontal line represents the median, asterisks indicate the mean value. Statistical analysis was performed by Student’s t-test and p ≤ 0.05 was considered significant.

Human immature MDC and CD14^+^ cells were obtained as previously reported [25] from buffy coats obtained from 6 different healthy donors and cultured at 10^6^ cells/ml with UV-inactivated bacteria at 1:10 cell ratio. The production of IL-1β, TNFα, and IL-6 was measured in supernatants collected after 16 hours of stimulation. **Fig 1** shows that the ST258-KP strains elicited a high production of TNFα, IL-6 and IL-1 β compared to not stimulated cultures. The figure also shows that the production of IL-1β was significantly lower in supernatants from monocytes and MDC cultured with KK207-1 compared with cells from KKBO-1. The production of IL-6 and TNFα induced by KKBO-1 was slightly higher compared to that induced by KK207-1 but statistical analysis did not reveal significant differences between the strains.

To confirm differences in the inflammatory potential between the two clades of ST258-KP we selected two additional strains with capsular phenotype of clade 2 and two additional strain of KPC-KP with capsular phenotype of clade 1 and used them as stimulus for monocytes and MDC for 6 hours. Table 2 shows that strains with capsular phenotype of clade 2 induced the production of higher amounts of IL-1β by monocytes and MDC compared with strains with capsular phenotype of clade 1. In contrast no significant differences in the production of TNFα and IL-6 were induced by bacterial cells from clade 2 or clade 1.

**Table 2:** IL-1β production by monocytes and MDC cultured with strains of *K*.*pneumoniae*-KPC with different capsular phenotype.

Monocytes	CPS _BO-4_			CPS _207–2_			
	KKBO-1	FIPP-1	MR190	06C07	KK207-1	01C03	CPS_BO-4_ vs CPS_207-2_
	**624.30**±55.61	**698.70**±181.71	**445.47**±3.13	**388.67**±43.25	**331.87**±50.88	**360.23**±54.53	**p = 0.048**
**MDC**	**629.13**±90.15	**666.33**±127.61	**582.33**±118.60	**313.33**±78.81	**296.60**±32.88	**361.47**±64.26	**p = 0.037**

Monocytes or MDC were cultured at 10^6^ cells/ml in RPMI with 10% FBS in the presence or absence (US) of UV-inactivated bacterial cells from strains KKBO1, FIPP-1, MR190 with capsular phenotype of clade 2 (cpsBO4) or with UV-inactivated bacterial cells from strains 06C07, KK207-1, 01C03 with capsular phenotype of clade 1 or with LPS (400 ng/ml). Cells were cultured with bacterial cells at 1:10 ratio. Conditioned media were collected after 6 hours of culture. IL-1β production was measured by Immunoplex array. Results of 3 different experiment are shown as mean ± SE, statistical analysis was performed by one-way ANOVA for mean comparison and p<0.05 was considered significant.

Notably the production of mature IL-1β is controlled by caspase-1-containing multi-protein complexes called “inflammasomes” [26–29] and at least two inflammasome pathways, NLRP3 and NLRC4 leading to caspase 1 activation [30], were described in infections with *K*. *pneumoniae* with different capsular phenotypes [31–33].

To investigate whether ST258 KP-KPC strains differently activate the NLRP3 and NLRC4 pathways we first studied the expression of the relative inflammasome proteins in monocytes and MDC from 3 different donors following culture with bacterial cells. Monocytes and MDC were cultured with live bacterial cells (1:1) for 6 hours and the expression of NLRP3 and NLRC4 was quantified by Western Blot analysis with specific antibodies. The results of these experiments showed that the expression of NLRP3 protein was differently affected by ST258-KP strains. In contrast NLRC4 expression was not induced by ST258-KP strains (data not shown)

**Fig 2:**
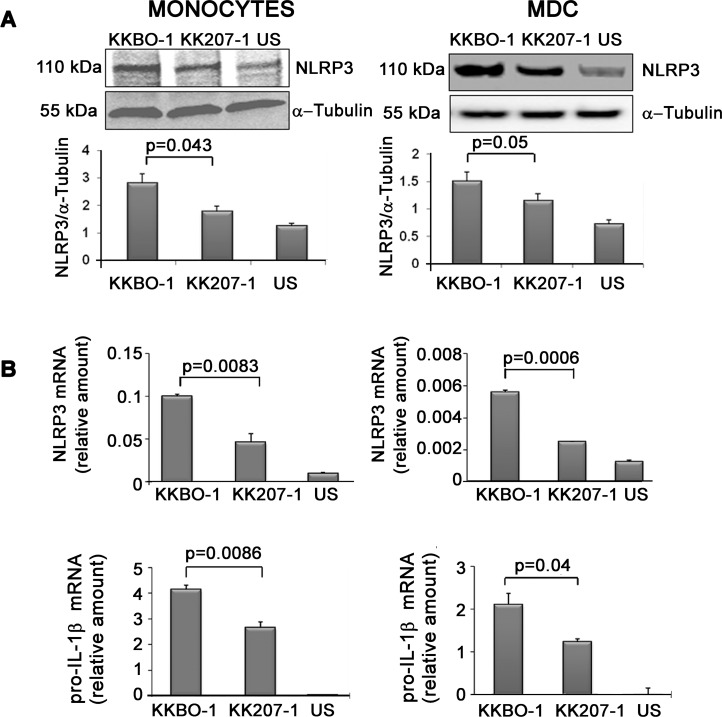
Effect of ST258 KP strains on NLRP3 expression. **Panel A: Effect of ST258 KP strains on NLRP3 protein expression.** Monocytes or MDC from 3 different donors were cultured at 10^6^ cells/ml in RPMI containing 10% FBS with live ST258 KP- strains at 1:1 ratio for 7 hours. At the end of incubation cells were lysed and analyzed by Western Blot. Results from one representative experiment are shown. The histogram below shows the results of densitometric analysis from the three different experiments (mean ± SE). **Panel B: Effect of ST258 KP strains on *NLRP3* and pro-*IL-1***β **(gene) expression.** Monocytes or MDC were cultured at 10^6^ cells/ml in the presence or absence of live ST258 KP strains for 4 hours at 1:1 cell ratio. Cells were lysed to obtain total RNA. The *NLRP3* and pro-*IL-1*β gene expression was evaluated by RT-PCR using specific primers. Results are expressed as mean ± SE of mRNA relative amounts (2^-ΔCT^) of experimental triplicates. Histograms show one representative experiment out of four performed. Statistical analysis was performed by Student *t*-test and p ≤ 0.05 was considered significant.

**Fig 2, panel A** shows that bacterial cells from KK207-1 induce NLRP3 expression in amounts significantly lower compared to cells from KKBO-1 in monocytes and in MDC.

The induction of NLRC4 protein by MDC and monocytes was low in these experimental conditions, and in any case they were not differently induced by the two ST258-KP strains. To confirm the differential expression of factors involved in the activation of NLRP3 inflammasome, MDC and monocytes were cultured with live bacterial cells in the same experimental conditions, and the amounts of mRNA for NLRP3 and pro-IL1β were quantified by RT-PCR.

**Fig 2, panel B** shows that live cells of KK207-1 activated NLRP3 and pro-IL-1β gene significantly less than KKBO-1, suggesting a lower ability of KK207-1 to prime monocytes and MDC for inflammasome activation compared to KKBO-1.

### Caspase-1 activation and cleavage of pro-IL-1β

**Fig 3:**
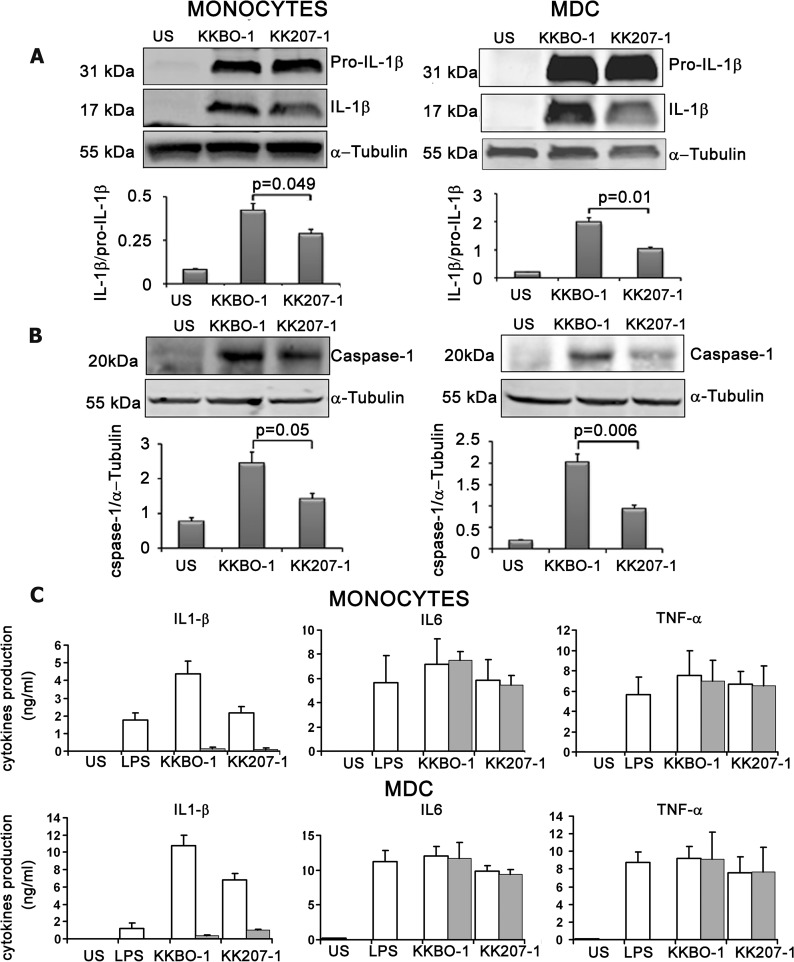
Effect of ST258 KP strains on NLRP3 in inflammasome activation. **Panel A/B: Effect of ST258-KP strains on pro-IL-1**β **cleavage and caspase-1 activation.** Monocytes or MDC from 3 different donors were cultured at 2x10^6^/ml with live ST258 KP strains for 7 hours at 1:1 cell ratio. Cells were lysed and immunoblotted with antibodies specific to IL-1β and caspase-1 (p20). Results from one representative experiment out of three performed are shown. Histograms show the results of densitometric analysis (mean ± SE) from three different experiments. **Panel C: Effect of glibenclamide on cytokine production by monocytes or MDC.** Monocytes or MDC from four different donors were cultured at 10^6^ cells/ml with live bacterial cells at 1:1 ratio in the presence (grey columns) or absence (white columns) of 100 μM glibenclamide. Cytokines were measured by Immunoplex array in conditioned media collected after 7 hours of culture.

As second step of our study we assessed the cleavage of caspase 1 and the production of mature IL-1β in lysates from monocytes and MDC cultured with live bacterial cells for 7 hours. **Fig 3, panel A** shows that KK207-1 induced the cleavage of pro-IL-1β in monocytes and MDC in amounts significantly lower compared to KKBO-1. **Fig 3, panel B** shows that KK207-1 also activated caspase 1 less than KKBO-1.

Finally, we treated monocytes and MDC with 100 μM glibenclamide, a sulfonylurea drug which inhibits NLRP3 activation but not NLRC4 activation [34]. After 30 min of treatment, cells were washed and cultured with live bacterial cells. Culture supernatants were collected for IL-1β measurement after 7 hours of culture. **Fig 3, panel C** shows that glibenclamide almost completely inhibited the bacterial cells-induced production of mature IL-1β in monocytes and in MDC. In contrast, it left unaffected the production of TNFα and IL-6.

Notably the activation of caspase-1 leads to programmed necrotic death of macrophages infected with viruses or bacteria [35]. Although cell death is not essential for caspase-1 mediated IL-1β release [36] we wanted to assess whether bacterial cells from the two ST258-KP strains differently activate necrotic death in macrophages. For this purpose we used PMA-differentiated THP-1 macrophages and cultured them with live bacterial cells at 1:1 cell ratio. Propidium iodide incorporation and LDH release was measured at different times by cytofluorimetric analysis and LDH assay respectively. We could detect both propidium iodide incorporation and LDH release after 16 hours of incubation. The results shown in **S1 Fig** revealed KKBO-1 as the strongest inducer of cellular death compared to KK207-1.

### Effects of purified capsular components on pathways involved in the production of inflammatory cytokines

To assess whether capsular composition of the two ST258-KP strains may affect pathways leading to inflammasome activation and/or cytokine production, we purified capsular polysaccharides from bacterial cells from KKBO-1 and KK207-1 as previously described [22] and studied their ability to affect pathways involved in the production of inflammatory cytokines in monocytes and MDC: in particular we studied

a) the activation of p38 MAPK and NF-κB (after 45 minutes of stimulation).

b) the expression of NLRP3 and pro-IL-1β (gene after 4 hours of stimulation)

c) the activation of caspase-1 and cleavage of pro-IL-1β in the mature form (after 7 hours of stimulation) in the presence or absence of ATP. For each experimental point, parallel experiments using live bacterial cells at 1:1 ratio as stimulus were performed.

**Fig 4:**
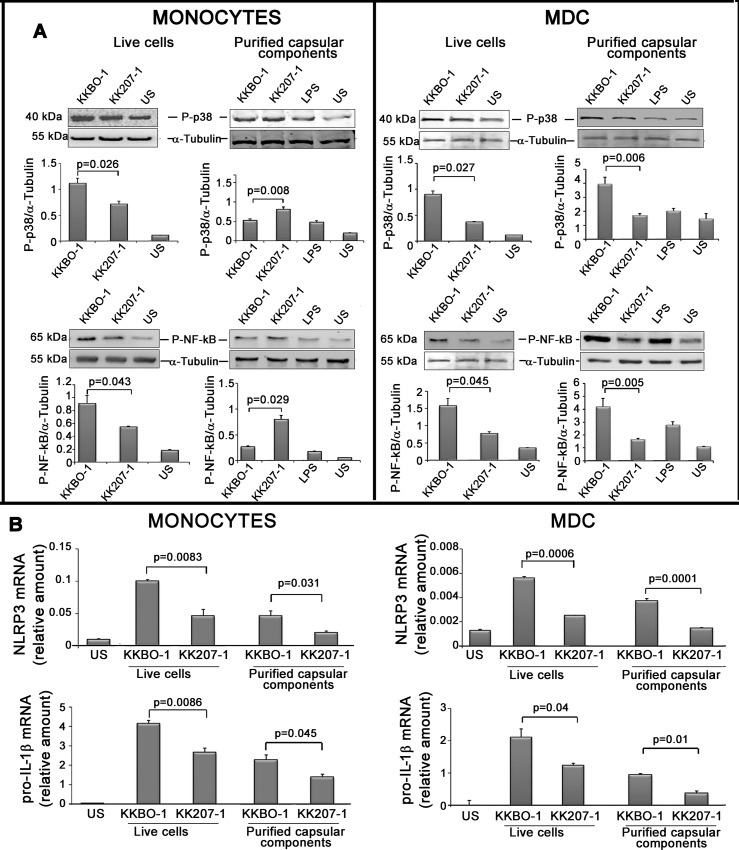
Effects of ST258 KP strains or purified capsular components from ST258 KP strains on pathway governing the *NLRP3* and pro-*IL-1*β genes. **Panel A: Effects of ST258 KP strains or purified capsular components from ST258 KP strains on p38 and NF-kB phosphorylation.** Monocytes or MDC were cultured at 10^6^ cells/ml with live ST258 KP strains (1:1 cell ratio) or with 5 μg/ml of purified capsular polysaccharide from ST258 KP strains for 45 minutes. Cells were lysed and phosphorylated p38 MAPK and NF-kB (p65) were detected by Western Blot analysis with specific antibodies. Results from one representative experiment out of three performed are shown. Histograms below show the ratio between the activated kinase and α-tubulin as revealed by densitometric analysis. Data from three independent experiments (mean ± SE) are reported. Statistical analysis was performed by Student’s *t*-test and p ≤ 0.05 was considered significant. **Panel B: Effects of ST258 KP strains or purified capsular components from ST258 KP strains on *NLRP3* and pro-*IL-1***β **gene expression.** Monocytes or MDC were cultured at 10^6^ cells/ml in the presence or absence of live bacterial cells (1:1 cell ratio) or 5 μg/ml of their purified capsular polysaccharides. After 4 hours cells were lysed to obtain total RNA and *NLRP3* and pro-*IL-1*β gene expression evaluated by RT-PCR using specific primers. Results are expressed as mean ± SE of mRNA relative amounts (2^-ΔCT^) of experimental triplicates. Histograms show results from one representative experiment out of four performed. Statistical analysis was performed by Student *t*-test and p ≤ 0.05 was considered significant.

**Fig 4** shows the effects of capsular polysaccharides on the phosphorylation of NF-κB, p38 MAPK, the expression of genes for NLRP3 and pro-IL-1β by monocytes and MDC. Capsular polysaccharides from KK207-1 were always less efficient in inducing these activities compared to capsular polysaccharides from KKBO-1. These data suggested that capsular composition may help bacterial cells to differently activate PRR pathways involved in the expression of inflammasome proteins.

**Fig 5:**
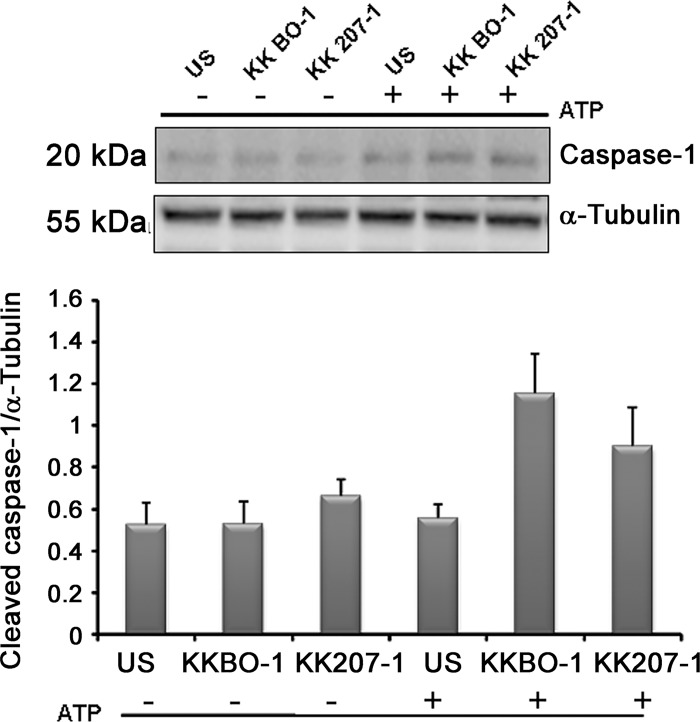
Effect of capsular polysaccharides from ST258 KP strains on caspase-1 activation. Monocytes were cultured at 2x10^6^/ml with purified 5 μg/ml of capsular polysaccharides from ST258-KP strains for 7 hours. Cells were lysed and immunoblotted with antibodies specific caspase-1 (p20) and tubulin. Results from one representative experiment out of three performed are shown. Histograms show the results of densitometric analysis (mean ± SE) from three different experiments.

While live bacterial cells were able to induce caspase 1 activation in monocytes and MDC (see **Fig 3**), **Fig 5** shows that the capsular preparations from KK207-1 or KKBO-1 failed to directly activate caspase-1 cleavage unless exogenous ATP was added to the culture.

## Discussion

Strains of ST258-KP can be segregated into two ST258 clades [4,11–14]. The major genetic divergence between these two clades is constituted by a 215-kb genomic region that includes the genes of the *cps* cluster. ST258-KP strains were shown to circumvent killing by human neutrophils [15,37] but no differences between strains with *cps*_BO-4_ (clade 2) or *cps*_*207-2*_ (clade 1) were revealed [11] suggesting that capsule switch does not confer to strains of the ST258-KP clades an additional benefit in terms of resistance to neutrophil phagocytosis. Although neutrophil phagocytosis is important in host defense against *K*. *pneumoniae* infection, different cells of innate and/or adaptive immunity also play relevant roles in infection clearance. MDC first sense bacterial cells once they penetrate mucosal barriers and, following interaction with bacterial ligands, they produce large quantities of inflammatory mediators involved in the recruitment of neutrophils, as well as in T-cell activation and differentiation [38,39]. Inflammatory monocytes, involved in the clearance of ST258-KP infection in mice, [40,41] also may produce high amounts of inflammatory (TNFα, IL-1β) or anti-inflammatory cytokines [42,43].

In this work, we studied strains of the two clades of the ST258 KP-KPC clonal lineage for their ability to activate pro-inflammatory functions in monocytes and myeloid dendritic cells isolated from normal immune competent donors. Our data showed that the two ST258-KP strains activated both monocytes and MDC to produce high amounts of pro-inflammatory cytokines (IL-1β, TNFα, and IL-6). Significant differences between the clades emerged only regard the production of IL-1β: Strains with capsular phenotype of clade 1 induced significant lower amounts of IL-1β production compared to strains with capsular phenotype of clade 2 both in monocytes and MDC while the production of TNFα and IL-6 was not differently affected by the two clades.

IL-1β is a potent inflammatory cytokine that induces the expression of adhesion molecules and regulates the influx of inflammatory cells [44]. The cytokine is also strongly involved in adaptive immunity since it potentiates IL-23 functions and Th17 differentiation [45–47]. Notably the production of IL-1β is tightly controlled at both transcriptional and post-translational levels. Synthesis of the immature precursor (pro-IL-1β) is initiated by the interaction of Pattern Recognition Receptors (PRR) with their ligands [48,49]. The cleavage of pro-IL-1β to mature form is controlled by caspase-1-containing multi-protein complexes called “inflammasomes”, each differentiated by unique activators, NLR/ALR family members, and caspase effectors [26–29]. At least two inflammasome pathways, NLRP3 and NLRC4, leading to caspase 1 activation [30], were described in *K*. *pneumoniae* infections [31–33].

We found that monocytes and MDC express higher amounts of NLRP3 protein when cultured with bacterial cells from ST258-KP strains. In contrast, scanty amounts of NLRC4 were detected in activated cells. Glibenclamide, a sulphonyl urea that inhibits the NLRP3-induced cleavage of pro-IL1β but not that induced by NLRC4 [34], almost completely blocked the release of mature IL-1β by ST258-KP strains. Although not formally demonstrated, these data suggested the involvement of NLRP3-inflammasome pathway in the production and release of mature IL-1β by ST258-KP strains. We observed that KK207-1 was much less efficient compared to KKBO-1 in priming monocytes and dendritic cells for inflammasome activation. Indeed, the transcription of genes for the inflammatory proteins NLRP3 and pro-IL-1β as well as the production of the corresponding proteins were significantly lower in cells cultured with KK207-1 compared to cells cultured with KKBO-1.

Notably in this “priming” phase, a multiplicity of interactions among bacterial ligands and the surface and cytosolic Pattern Recognition Receptors (PRRs) expressed by innate cells occur. The strength of interactions as well as the combination of activating and inhibitory pathways finally control the amounts NLRP3 and pro-IL-1β transcripts. Both NF-κB and MAPK phosphorylation [50–53] are involved in transcription and stabilization of mRNAs for NLRP3 and pro-IL1β. We found that KK207-1 was less efficient in inducing NF-κB and p38MAPK phosphorylation compared to KKBO-1. These differences were low but statistically relevant and were detected both on monocytes and MDC. Although similar pathways regulate the transcription of genes for multiple inflammatory cytokines significant differences between the two clades emerged only for the production of IL-1β. We explain these data with the consideration that bacterial factors affect both the transcriptional and post-translational level of regulation of IL-1β production.

Indeed, we showed significant differences in the ability of ST258-KP strains to induce caspase 1 activation and pro-IL-1β cleavage ether in monocytes and MDC. In each case KK207-1 was less efficient compared to KKBO-1. Although experiments using NLRP3 siRNA or NLRP3-KO cell lines will definitely establish the involvement of this pathway in IL-1β production by ST258 KP, our data strongly suggest that, compared to KKBO-1, KK207-1 might partially escape this inflammasome recognition. According with this hypothesis is also the lower level of inflammatory cell death induced by KK207-1 compared to KKBO-1 in a human macrophage cell line.

The factor/s involved in the activation or in the attenuation of the process still remain to be defined. Indeed capsular polysaccharides, which represent the main divergence between ST258-KP clades, may interact with selected PRR including those governing the availability of inflammasome proteins, being the strength of interactions dependent on polysaccharide composition. This hypothesis is suggested by the observation that capsular polysaccharides from KK207-1 are less efficient in activating the phosphorylation of NF-κB and p38MAPK compared to those purified from KKBO-1. Based on these data, we speculate that bacterial cells from KK207-1 benefit from the rearrangement of capsular composition to partially escape innate recognition and to interfere with upstream signaling pathway governing inflammasome activation. In other words, the clade evolved from ST258-KP could be better adapted to escape the innate immune response. Further studies are needed to establish whether the “adapted” subclones are also endowed with a more aggressive phenotype as suggested by data obtained in *Galleria mellonella* system [54] and, recently, by surveillance data from a multicenter consortium in USA [55].

## Supporting Information

S1 Fig Activation of necrotic death in THP1 cells by bacterial cells from KKBO1 and KK207-1 strains. PMA-differentiated THP-1 macrophages were cultured with live bacterial cells from KKBO1 and KK207-1 strains at 1:1 cell ratio. The histograms show the incorporation of propidium iodide and the release of LDH induced by bacterial cells after 16 hours of incubation. Statistical analysis was performed by Student *t*-test and p ≤ 0.05 was considered significant.
(JPG)
