# Supplementary figures and images for: Differences in Inflammatory Response Induced by Two Representatives of Clades of the Pandemic ST258 Klebsiella pneumoniae Clonal Lineage Producing KPC-Type Carbapenemases
Source: PLoS One. 2017 Jan 12;12(1):e0170125. doi: 10.1371/journal.pone.0170125 (PMC5231394; doi:10.1371/journal.pone.0170125)

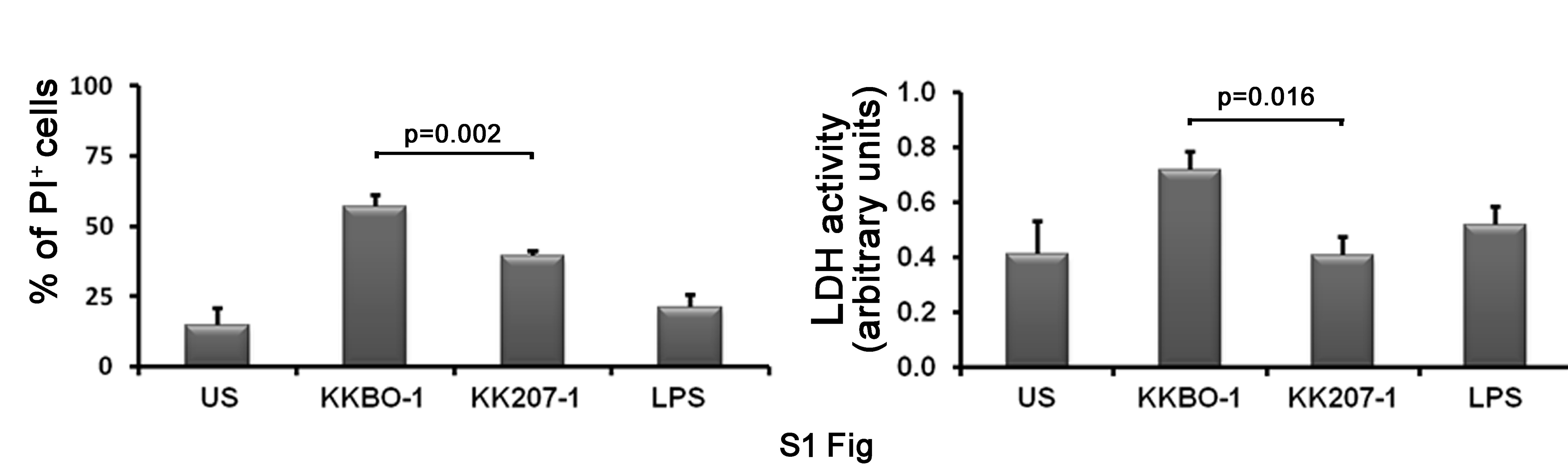

Supplement: S1 Fig — PMA-differentiated THP-1 macrophages were cultured with live bacterial cells from KKBO1 and KK207-1 strains at 1:1 cell ratio. The histograms show the incorporation of propidium iodide and the release of LDH induced by bacterial cells after 16 hours of incubation. Statistical analysis was performed by Student t-test and p ≤ 0.05 was considered significant. (JPG) [file pone.0170125.s001.jpg]
